# Maxillary 1^st^ molar with three canals in mesiobuccal root

**DOI:** 10.11604/pamj.2022.42.125.34812

**Published:** 2022-06-16

**Authors:** Akash Jitender Sibal, Shriya Ravindra Singi

**Affiliations:** 1 Department of Conservative Dentistry and Endodontics, Sharad Pawar Dental College and Hospital, Datta Meghe Institute of Medical Sciences (Deemed to be university), Wardha, India

**Keywords:** Maxillary 1^st^ molar, mesiobuccal canal, obturation

## Image in medicine

A comprehensive knowledge of the root and canal configuration is a prerequisite before initiating any endodontic procedures. A 32-year-old male reported to the department of conservative dentistry and endodontics with a complaint of pain associated with tooth #16 (maxillary first molar). Patient was experiencing pain since more than 25 days and was on medication for symptomatic relief. Initial clinical examination revealed the probability of periapical or phoenix abscess as the tenderness on percussion test was positive and the pulp showed no response during the pulp vitality test. However, the intraoral periapical radiograph depicted distoproximal caries but no signs of abscess or radiolucency in the periapical region associated with #16, and the patient was diagnosed with symptomatic irreversible pulpitis. Root canal therapy was planned for the patient. During access cavity preparation an unusual occurrence of three canals in the mesiobuccal root of #16 was found. So, there was a total of five canals in the three rooted #16. All canals were biomechanically prepared and obturated to achieve a hermetic seal. The current case presentation emphasizes on the high possibility of unexpected variations in the root and its canal morphology. Awareness regarding such variations will minimize the intra-operative complications during root canal therapy and will avoid root canal failure.

**Figure 1:**
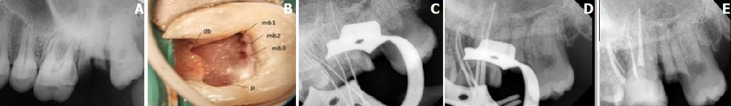
pre-operative radiograph showing distoproximal caries (A); access opening showing threw canal orifices in mesiobuccal root (B); working length determination (C); evaluation of master cone fit (D); obturation with gutta percha (E)

